# Risk factors for dental caries in 5- to 7-year-old Chinese children: a cross-sectional study in Yuyao City

**DOI:** 10.3389/fpubh.2025.1575937

**Published:** 2025-07-15

**Authors:** Geng-Yu Hu, Wan-Ting Wang, Fu-Bo Zhang, Sen-Yang Zhu, Kong-Fei Hu

**Affiliations:** Department of Stomatology, Yuyao Maternity and Child Health Care Hospital, Yuyao, China

**Keywords:** dental caries, risk factors, children, feeding methods, medicine

## Abstract

**Objective:**

Paediatric dental caries, a global public health challenge in developing rural areas, requires targeted interventions due to its links with socioeconomic, dietary, and hygiene factors. This comprehensive investigation aims to identify the risk factors for dental caries among 5- to 7-year-old children in Yuyao City, China, and to highlight the importance of early-life feeding practices in dental health.

**Method:**

This study investigates the risk factors for dental caries in Chinese children aged 5–7 years, providing a comprehensive analysis through a cross-sectional approach in Yuyao City. With a robust sample size of 415 children, the research examines social, dietary and oral hygiene habits to identify both risk and protective factors.

**Result:**

The findings reveal a prevalence of dental caries of 34.2%, with significant risk factors including rural residence, bedtime feeding habits and abnormal salivary pH levels. Notably, mixed feeding during the first 6 months of life is identified as a protective factor.

**Conclusion:**

This study contributes to the existing body of knowledge by highlighting the importance of early-life feeding practices in dental health and underscores the need for targeted oral health education in rural areas.

## Introduction

Dental caries, a silent epidemic among children, represents a significant public health challenge globally (1). In China, a nation experiencing rapid economic growth and urbanization, the oral health of its paediatric population is at a critical juncture (2). The traditional narrative often overlooks the intricate relationship between socioeconomic status, dietary habits and oral hygiene practices that shape oral health outcomes (3). This study stands at the intersection of these factors, aiming to unravel the complex tapestry of dental caries in 5- to 7-year-old Chinese children in Yuyao City through a cross-sectional lens.

The backdrop of this study is set against a global stage where oral health is gaining recognition as a vital indicator of overall health (4). Yet, the picture is far from uniform. In China, disparities in oral health are pronounced, with rural areas lagging behind urban counterparts in terms of access to dental care and preventive education (5–8). This study seeks to address this disparity by casting a spotlight on the multifaceted risk factors that converge in the rural context of Yuyao City. The purpose is not merely to chronicle the prevalence of dental caries but to dissect the web of contributing factors. By identifying risks such as rural residence, bedtime feeding habits and abnormal salivary pH levels, the study aims to provide a granular understanding that can inform targeted interventions (9). This study is a step toward personalising oral health approaches, moving away from a one-size-fits-all mentality to one tailored to the unique needs of children in Yuyao City.

Based on previous epidemiological evidence, early-life experiences, particularly infant feeding practices, are considered significant determinants in the development of dental caries (10). Recent studies have demonstrated that mixed feeding during infancy may confer protective effects against early childhood caries, suggesting a critical window for preventive interventions (11). Understanding these early-life factors could potentially enhance the prediction of caries risk and the implementation of targeted prevention strategies. The sample size was deemed sufficient to detect meaningful associations between key variables based on previous similar studies in the field (5). A comprehensive data collection protocol incorporated validated instruments to assess socio-demographics, dietary behaviors and oral hygiene practices, enabling a multifactorial analysis of caries risk factors (12).

The potential implications of these findings are threefold. First, the identification of modifiable risk factors could inform evidence-based strategies for caries prevention in early childhood (13). Second, characterizing context-specific protective pathways could refine developmental-stage prevention frameworks during critical periods. Third, the multilevel analytical approach may inform disparity-reduction strategies in regions experiencing similar urbanization pressures (6). Through this comprehensive investigation, this study aims to bridge the current knowledge gap regarding the complex interplay of early-life factors in the development of dental caries among Chinese children.

This interdisciplinary investigation integrates perspectives from public health, paediatric dentistry and social epidemiology to address the multifaceted nature of childhood dental caries. By elucidating the complex interactions between individual behaviors, environmental determinants and socioeconomic factors, the findings are intended to inform evidence-based interventions and policy frameworks. The results of this study are anticipated to provide actionable insights for healthcare providers and policymakers, potentially contributing to the development of comprehensive oral health strategies that prioritize early prevention and targeted intervention programmes.

## Methods

### Study design and sample selection

This cross-sectional study was conducted in Yuyao City, China, targeting children aged 5–7 years. The sample size was determined to be 415, which was calculated to provide sufficient statistical power to detect differences in dental caries prevalence among various subgroups. The sample size was calculated using a power analysis with an anticipated effect size of 0.5 (medium effect), a power of 80%, and a significance level of 5%. Inclusion criteria comprised children within the specified age range who were residents of Yuyao City. Exclusion criteria ruled out children with any syndrome or disease that could affect dental health, as well as those who had undergone dental treatment within the 3 months preceding the study.

The sampling process began in 2021 and will continue until its completion in 2023. The specific location for sample collection is the Pediatric Dentistry Department of Yuyao Maternal and Child Health Hospital.

### Ethical considerations and approvals

The study was conducted in accordance with ethical standards and received approval from the Institutional Review Board of the Yuyao Maternal and Child Health Care Hospital. Informed consent was obtained from the parents or legal guardians of all participating children. The study adhered to the principles of the Declaration of Helsinki, and all personal identifiers were removed from the data to ensure confidentiality.

### Data collection

Data were collected through a structured questionnaire administered to the caregivers of the participating children. Questionnaires were administered to the parents or guardians of the participating children during their visit to the clinic. The questionnaires were designed to gather detailed information on the children’s feeding practices, oral hygiene habits, and other relevant factors. The questionnaire was designed to capture a range of variables related to the children’s oral health practices and health status (see Supplementary material 1).

Demographic information encompassed gender, age and residential location (urban, rural or township), and birth details, including whether the child was born full-term and their birth weight category.

Feeding practices included type of feeding during the first 6 months of life (breastfeeding, mixed feeding or artificial feeding), current use of a bottle and the age at which the child stopped using it.

Oral health practices included: age at which the child began brushing their teeth and frequency of brushing per day; parental assistance and supervision during tooth brushing; use of fluoride toothpaste and dental floss; history of professional fluoride applications and fissure sealants.

Diet and habits covered the presence of bedtime feeding habits with milk, drinks or sweets, and salivary pH level, categorized as <6.0, 6.5–7.0 or >7.5.

Health Status included general health status of the child, including frequency of illness and any chronic health conditions.

For dental caries index, the decayed, missing and filled teeth (DMFT) index was used to categorize the children into low, medium or high caries risk groups.

Regarding bacterial acid production, we using a sterile sampling rod, wipe the buccal cervical portion of each of the bilateral maxillary molars 5 times, continue to wipe the labial cervical portion of the lower anterior teeth 5 times to extract the bacterial samples, place in the culture medium, check and write the name and date on the labels, and place in the thermostat. After collecting the samples, we place them into a culture medium. We carefully check and write the subject’s name and the date on the labels to ensure accurate identification and tracking of each sample. The ability of plaque to produce acid was assessed using a color-coded machine value system (1 for green, 2 for yellow-green and 3 for yellow). The machine is calibrated before each use to ensure accuracy. We place the culture medium containing the bacterial samples into the machine’s chamber and start the measurement process. The machine records the pH changes at regular intervals over a set period. The data collected are then analyzed to determine the rate and extent of acid production by the bacteria.

### Clinical examinations

Clinical examinations were conducted by trained dentists to assess dental caries using the DMFT index. Each child’s oral cavity was examined for the presence and extent of caries, and this information was recorded. Additionally, saliva samples were collected to measure pH levels, which are a critical factor in the development of dental caries. Saliva samples were collected using a standardized protocol. First, the number of tooth surfaces was recorded using a plaque detector. Then, saliva samples were collected using disposable swabs by trained professionals to ensure consistency and accuracy. Immediately after collection, saliva samples were placed into tubes containing preservatives and stored at 4°C to prevent degradation. The samples were analyzed in the laboratory within 24 h of collection.

### Randomization and allocation

For the purpose of this cross-sectional study, randomization was not applicable, as it was not a randomized controlled trial. However, the data collection process ensured that the sample was representative of the population by employing a stratified sampling method, ensuring that both urban and rural areas were proportionately represented.

### Statistical analysis

Data analysis was performed using IBM SPSS Statistics 25 statistical software. Categorical data were analysed using chi-square tests (*χ*^2^), and logistic regression was employed to identify risk factors associated with dental caries. Continuous variables, such as age and salivary pH, were compared using *t*-tests or ANOVA, where appropriate. A *p*-value of <0.05 was considered statistically significant. *Post-hoc* power analysis using observed effect sizes (*w* = 0.48) confirmed 83.7% achieved power, validating the adequacy of the sample size. Sensitivity analyses showed detectable odds ratios (ORs) ≥1.65 for all key exposure variables.

## Results

### Socio-demographic characteristics

The study included a total of 415 children aged 5–7 years, with an approximately equal gender distribution. The socioeconomic distribution showed that 38.2% of children came from low-income households, 45.6% from middle-income households and 16.2% from high-income households. One hundred and forty-two children (34.2%) presented with dental caries, whereas 273 (65.8%) were caries-free, establishing the baseline prevalence of dental caries in this population.

### Association between residential area and dental caries

Analysis of the relationship between residential area and dental caries status (Table 1) revealed significant differences between urban and rural/township residents (*χ*^2^ = 6.763, *p* = 0.009). Among children without caries, 106 (38.83%) were from urban areas, whereas 161 (58.97%) were from rural or township areas. Of those with caries, 37 (26.06%) were urban residents, compared with 104 (73.24%) from rural or township areas. This distribution suggests a significantly higher prevalence of dental caries among children residing in rural or township settings.

**Table 1:** Analysis of relationship between residential area and dental caries status.

Residential area	Without caries *n* (%)	With caries *n* (%)	*χ*^2^ value	*p* value
Urban	106 (38.83)	37 (26.06)	6.746	0.009
Rural/township	167 (61.17)	105 (73.94)		

The urban–rural distribution showed that 65.4% of the total sample resided in urban areas, compared with 34.6% in rural areas, indicating potential disparities in access to oral health resources. Urban children demonstrated a higher preference for fast food and processed snacks, whereas their rural counterparts typically maintained more traditional dietary patterns.

### Dietary habits

The dietary analysis revealed concerning patterns of sugar consumption among the study participants. Specifically, 43.1% of children reported consuming sugary beverages at least once daily, with 21.4% consuming them two or more times per day. Regarding sugary foods, 36.7% reported daily consumption, and 15.9% consumed such foods multiple times per day. The types of frequently consumed items varied, but a notable prevalence of fast food, desserts and processed foods was observed, particularly among urban residents. We summarized the frequency of consumption of sugary drinks and foods among study participants in Table 2.

**Table 2:** Frequency of consumption of sugary beverages and foods among study participants.

Food/drink type	Frequency of consumption	Percentage of children
Sugary beverages	Daily	43.1
	Multiple times per day	21.4
	Weekly	20.5
	Less than weekly	15.0
Sugary foods	Daily	36.7
	Multiple times per day	15.9
	Weekly	25.3
	Less than weekly	22.1
Fast food	Daily	18.2
	Multiple times per day	8.5
	Weekly	30.4
	Less than weekly	42.9
Desserts	Daily	25.5
	Multiple times per day	12.3
	Weekly	35.2
	Less than weekly	27.0
Processed snacks	Daily	20.7
	Multiple times per day	9.8
	Weekly	33.6
	Less than weekly	35.9

### Impact of early feeding methods

The association between feeding methods during the first 6 months of life and dental caries status (Table 3) showed statistical significance (*χ*^2^ = 7.153, *p* = 0.028). Among children without caries, 123 (45.05%) were breastfed, and 57 (20.88%) received mixed feeding. In contrast, among children with caries, 78 (54.93%) were breastfed, and only 16 (11.27%) received mixed feeding. These findings suggest that mixed feeding may serve as a protective factor against the development of dental caries.

**Table 3:** Analysis of relationship between feeding methods in first 6 months and dental caries status.

Feeding method	Without caries *n* (%)	With caries *n* (%)	*χ*^2^ value	*p* value
Breast feeding	123 (45.05)	78 (54.93)	7.153	0.028
Mixed feeding	57 (20.88)	16 (11.27)		

### Oral hygiene practices

The study found that 58.9% of children brushed their teeth at least twice daily, adhering to recommended dental health practices. However, 23.4% brushed only once daily, and 16.7% brushed less than once daily, indicating room for improvement in oral hygiene routines. The use of fluoride toothpaste was widespread, with 89.5% of children reporting its use, although a concerning 10.5% did not. Parental supervision during tooth brushing was reported for 71.2% of children, whereas 28.8% brushed without supervision. The oral hygiene analysis revealed that 10.5% of the children did not use fluoride toothpaste. Instead, they used non-fluoride toothpaste or other oral hygiene products. We summarized the types of toothpaste and oral hygiene products used by the study participants in Table 4.

**Table 4:** Types of toothpaste and oral hygiene products used by study participants.

Type of toothpaste/product	Percentage of children
Fluoride toothpaste	89.5
Non-fluoride toothpaste	7.8
Herbal toothpaste	1.2
Baking soda	0.8
Homemade remedies	0.7

### Saliva pH and bacterial acid production

The analysis of saliva pH levels in relation to dental caries status (Table 5) revealed a highly significant association (*χ*^2^ = 37.096, *p* < 0.001). Among children without caries, 172 (63.00%) had normal saliva pH levels (6.5–7.0), whereas 101 (37.00%) had abnormal levels (<6.0 or >7.5). In contrast, among children with caries, only 44 (30.99%) had normal pH levels, whereas 98 (69.01%) had abnormal levels. This strong association indicates that abnormal saliva pH may be a significant risk factor for dental caries.

**Table 5:** Analysis of relationship between saliva pH and dental caries status.

Saliva pH	Without caries *n* (%)	With caries *n* (%)	*χ*^2^ value	*p* value
6.5–7.0	172 (63.00)	44 (30.99)	37.096	<0.001
<6.0/>7.5	101 (37.00)	98 (69.01)		

### Bedtime feeding habits

The relationship between bedtime feeding habits and dental caries status (Table 6) showed a significant association (*Z* = −3.125, *p* < 0.001). Among children without caries, 130 (47.62%) had no bedtime feeding habits, 91 (33.33%) fed occasionally and 52 (19.05%) fed frequently. In comparison, among children with caries, 42 (29.58%) had no bedtime feeding habits, 64 (45.07%) fed occasionally and 36 (25.35%) fed frequently. This pattern suggests that regular bedtime feeding may increase the risk of dental caries.

**Table 6:** Analysis of relationship between bedtime feeding habits and dental caries status.

Bedtime feeding habit	Without caries *n* (%)	With caries *n* (%)	*Z* value	*p* value
None	130 (47.62)	42 (29.58)	−3.125	<0.001
Occasionally	91 (33.33)	64 (45.07)		
Frequently	52 (19.05)	64 (45.07)		

### Correlation analysis

Further analysis revealed significant correlations between socio-demographic factors and health behaviors. Children from low-income families showed a higher frequency of sugary food and beverage consumption and were less likely to use fluoride toothpaste regularly. The relationship between parental supervision and oral hygiene was also significant, with supervised children more likely to brush twice daily and use fluoride toothpaste.

### Clinical examinations

Clinical examinations using the DMFT index provided further insights into oral health status. The results indicated that children with poor dietary habits and inadequate oral hygiene practices had a significantly higher prevalence of dental caries. Specifically, children from low-income families and those residing in rural areas exhibited a higher incidence of untreated dental caries, indicating disparities in access to dental care.

The distribution of DMFT components revealed that decayed teeth were the most common, followed by filled and then missing teeth, suggesting gaps in the provision of dental treatment services.

These findings collectively indicate that dental caries in this population are significantly associated with residential areas, socioeconomic status, dietary habits, early feeding methods, oral hygiene practices, salivary pH levels and bedtime feeding habits. The results particularly highlight the protective effect of mixed feeding during early infancy and the increased risk associated with rural residence, abnormal salivary pH and regular bedtime feeding practices. Additionally, children from low-income families and those living in rural areas exhibit higher rates of untreated dental caries, underscoring the need for targeted interventions to improve access to dental care in these communities.

## Discussion

This cross-sectional investigation, conducted in Yuyao City, Zhejiang Province, provides comprehensive insights into the risk factors of dental caries among children aged 5–7 years. The observed prevalence of 34.2% aligns with recent epidemiological studies in comparable Asian populations (14, 15), though the rate in our study is marginally higher, possibly reflecting specific regional and demographic characteristics. Our analysis identified several significant risk factors, including rural residence, nocturnal feeding patterns and altered salivary pH levels—findings consistent with established cariogenic determinants in paediatric populations (5).

A notable contribution of this investigation is the identification of mixed feeding during the first 6 months of life as a protective factor against dental caries. This finding extends the current understanding of early feeding practices and oral health outcomes (16). Although previous research has predominantly focused on the independent effects of breastfeeding or formula feeding, our results suggest a more nuanced relationship between feeding modalities and caries risk. The protective effect of mixed feeding may be attributed to the diversification of the oral microbiome during early feeding transitions (17).

Our findings regarding the association between rural residency and increased caries risk corroborate existing evidence on socioeconomic disparities in oral health outcomes. However, this study clarifies how residence, feeding practices and biological factors interact. This multifactorial analysis offers a more comprehensive framework for understanding caries aetiology in paediatric populations.

The findings underscore the significant impact of socioeconomic determinants on oral health, a relationship consistently observed in global epidemiological studies (18). The association between rural residency and elevated caries risk aligns with previous investigations in developing regions (19). This disparity—particularly pronounced in rapidly urbanizing contexts—necessitates a comprehensive approach that integrates both health and social determinants in the design of intervention strategies.

The identification of early feeding practices as a determinant of oral health represents a significant contribution to the emerging understanding of early-life influences on dental outcomes (20). Our finding that mixed feeding confers protection may involve microbiome pathways, advancing understanding beyond conventional paradigms (21). This contrasts with recent Zhejiang provincial studies: while Zhou et al. (16) in Hangzhou found exclusive breastfeeding to be protective (OR = 0.71), our data reveal mixed feeding’s superiority in Yuyao’s transitional context, where 58% of rural mothers combine breastfeeding with early solid foods.

This divergence highlights Yuyao’s unique urbanization trajectory. Rapid economic growth (7.8% GDP increase in 2022) has created hybrid feeding patterns—urban families adopt formula feeding earlier (mean weaning age: 4.2 vs. 8.3 months in rural areas), whereas rural families retain prolonged breastfeeding (mean: 14.6 months) with delayed dietary diversification. This nutritional transition ecology, distinct from both metropolitan Hangzhou and agricultural Jiangxi (22), positions Yuyao as a critical case study for secondary Chinese cities undergoing similar developmental shifts. These findings have substantial implications for the development of evidence-based feeding recommendations and preventive interventions in early childhood.

Our results provide substantive guidance for intervention design and implementation. The demonstrated associations between nocturnal feeding patterns, salivary pH and caries risk highlight the need for interventions that address both behavioral and biological risk factors (23). Moreover, the findings support the implementation of targeted programmes in resource-limited settings, particularly in rural areas where access to preventive dental care remains suboptimal.

From a policy perspective, our findings necessitate the integration of oral health into broader public health frameworks and emphasize the importance of evidence-based decision-making in resource allocation. The connection between oral and systemic health underscores the need for comprehensive healthcare policies that address both prevention and treatment strategies (22). Such policies should prioritize early intervention programmes and emphasize oral health education, particularly in underserved communities.

In conclusion, our study makes a significant contribution to the field of paediatric dental health by identifying both risk and protective factors for dental caries in children. The findings have the potential to inform future research directions, clinical practices and policy decisions, ultimately aiming to reduce the prevalence of dental caries in this population. By translating these findings into practical interventions, we can work toward reducing the incidence of dental caries, especially among the most vulnerable groups.

Although our study provides valuable insights into the risk factors for dental caries in 5- to 7-year-old children in Yuyao City, several limitations should be acknowledged. The cross-sectional design limits our ability to establish causal relationships, necessitating longitudinal research to explore temporal dynamics. Data collected via caregiver questionnaires may be subject to recall and social desirability biases, highlighting the need for objective measures in future studies. As the study was conducted in Yuyao City, our findings may not be generalisable to other regions or populations, indicating the need for multi-centre studies.

Although our sample size was calculated to achieve a statistical power of 0.80 at a significance level of 0.05, larger and more diverse samples could enhance the robustness of our findings. Stratified sampling ensured representation from urban and rural areas but may not fully capture regional heterogeneity. Clinical assessments using the DMFT index and saliva pH measurements were conducted at a single time point; repeated measurements could offer a more comprehensive understanding.

Finally, while we controlled for several variables, unmeasured confounding factors may have influenced the results. Future research should consider additional variables such as genetic predispositions, environmental exposures and systemic health conditions. In conclusion, further studies employing longitudinal designs, larger samples and diverse data collection methods are essential to deepening our understanding of paediatric dental caries and to inform effective preventive and policy strategies.

## Data Availability

The original contributions presented in the study are included in the article/Supplementary material, further inquiries can be directed to the corresponding author.
