# Supplementary material for: Risk factors for dental caries in 5- to 7-year-old Chinese children: a cross-sectional study in Yuyao City
Source: Front Public Health. 2025 Jul 15;13:1575937. doi: 10.3389/fpubh.2025.1575937 (PMC12303984; doi:10.3389/fpubh.2025.1575937)
Supplement: Supplementary file 1 [file Data_Sheet_1.docx]

**Evaluation questionnaire for Risk Factors for Dental Caries**

1、Are you the child’s ?

○Father ○Mother

2、What is the sex of the child

○male ○female

3、Where is your family located?

○Urban ○Rural

4、Does the child's father have dental caries?

○Yes ○No ○Don't know

5、Highest level of education of the child's mother?

○Below junior high school ○High school/junior college

○College and bachelor's degree ○achelor's degree or above

6、How often does the child's mother currently brush her teeth each day?

○2 or more times a day ○Once a day ○Occasionally/no brushing

7、Do mothers of children floss?

○Yes ○No

8、Does the child's mother have dental caries?

○Yes ○No ○Don't know

9、Is your child an only child?

○Yes ○No

10、Does your child have a habit of eating milk, drinks, or desserts after brushing his/her teeth at bedtime?

○No ○Occasionally ○Frequently

11、How often does your child usually eat the following sugary foods or beverages (sweet snacks such as cookies, cakes, breads, candies, chocolates, sweetened beverages such as sugar water, honey water, carbonated beverages such as cola, fruit juices such as orange juice, apple juice, non-freshly squeezed juices such as lemonade, and sweetened milk/yogurt/soymilk)?

○Occasionally/never ○1~3 times per month

○1~6 times per week ○1 time or more per day

12、Did your child start brushing his/her teeth after the eruption of the first milk tooth?

○No ○Yes ○Don't remember

13、How many times a day does your child currently brush his/her teeth?

○2 or more times a day ○1 time a day ○No brushing/occasional brushing

14、Do you help your child floss?

○Frequently ○Occasionally ○Never

15、What was your child's age when you first took him/her for an oral exam?

○0~6 months ○7~12 months ○1~2 years old

○2~3 years old ○Never had an oral examination

1. Has your child received professional fluoridation (fluoride foam, fluoride protective paint, fluoride gel, etc.)?
    ○Regularly ○Occasionally ○Never

17、Has your child had his/her fissure closed?

○Yes ○No

18、Do you think your child needs to be treated if caries occurs?

○Must be treated ○Depends on the child's condition, if he/she can cooperate, then treat him/her

○No treatment is needed because the child will have new teeth in the future.
